# An ontology for cell types

**DOI:** 10.1186/gb-2005-6-2-r21

**Published:** 2005-01-14

**Authors:** Jonathan Bard, Seung Y Rhee, Michael Ashburner

**Affiliations:** 1 Department of Biomedical Sciences, Hugh Robson Building, University of Edinburgh, Edinburgh, EH8 9XD, UK; 2 Department of Plant Biology, Carnegie Institution of Washington, Stanford, CA 93405, USA; 3 Department of Genetics, University of Cambridge, Cambridge, CB2 3EH, UK; 4 EMBL-European Bioinformatics Institute, Hinxton, Cambridge, CB10 1SD, UK

## Abstract

We describe an ontology for cell types that covers the prokaryotic, fungal, animal and plant worlds. It includes over 680 cell types. These cell types are classified under several generic categories and are organized as a directed acyclic graph. The ontology is available in the formats adopted by the Open Biological Ontologies umbrella and is designed to be used in the context of model organism genome and other biological databases. The ontology is freely available at  and can be viewed using standard ontology visualization tools such as OBO-Edit and COBrA.

## Abstract 2

An ontology for cell types that covers the prokaryotic, fungal, animal and plant worlds is described. It includes over 680 cell types. These cell types are classified under several generic categories and are organized as a directed acyclic graph.

## Background

One of the most challenging problems now facing the model organism databases is the formal description of phenotypic data. While some databases, for example those for mouse (*Mus musculus*) [1], corn (*Zea mays*) [2] and fruit fly (*Drosophila melanogaster*) [3], include a rich heritage of data describing the phenotypes of mutants, and some progress is being made to bring these data into a well structured computable representation [3-5], the annotation of these phenotypes is hampered by a lack of structured information describing a variety of other biological objects, including cell types. A structured vocabulary of cell types is also required by databases for the description of other biological objects, such as gene-expression data. In addition, using the same concepts for the description of these data in all of these databases would facilitate interoperability among them.

To address these needs, we have developed an ontology that describes the cell types of the major model organisms, both animal and plant. Its use will allow a biologist to query a single database with such questions as: list all of the cell types in mouse that express the *Notch *gene and all of the cell types in *Drosophila *and *Caenorhabditis elegans *that express the closest homolog of this gene; list all of the genes in mouse, rat, human and zebrafish that are expressed in the cell type Schwann_cell; CL:0000218; list all of the genes in *D. melanogaster *and *C. elegans *that have a mutant phenotype in the cell types that develop from the cell type myoblast; CL:0000056. The use of the cell ontology will thereby promote the *de facto *integration of data from diverse databases.

Since the development of the Gene Ontology (GO) for the annotation of attributes of gene products [6], many ontologies have been developed in the model organism informatics community. Several of these are available, in a choice of common formats, from the Open Biological Ontologies (OBO) site [7]. They include comprehensive developmental and anatomical ontologies for many model organisms (for example, mouse, *Drosophila*, *Arabidopsis thaliana *and *C. elegans*), and ontologies for mouse pathology and human disease.

There are several other ontologies that include cell types such as Systematized Nomenclature of Medicine (SNOMED) [8], the Foundational Model of Anatomy (FMA) [9], the anatomy ontologies used in model organism databases at the OBO site [7], vocabularies used by the resources that hold cell lines such as the American Type Cell Collection (ATCC) or the European Collection of Cell Cultures (ECACC) [10,11], and others [12,13]. Our approach for handling cell types differs from that adopted by these resources. First, SNOMED, FMA and the species-specific anatomy ontologies explicitly assume that the cell types they include are associated with one particular organism. Their identifiers cannot therefore be used to annotate cell types from other organisms, even if these cell types are essentially identical to those in the organism-specific ontologies. Second, these resources, together with those that hold cell lines (for example, ECACC and ATCC), tend to define cell types as constituents of tissues rather than provide phenotypic information about their attributes - the knowledge that they encapsulate is severely limited. Third, some ontologies do not have publicly available identifiers for each term; hence they cannot be used for general annotation [10,11]. The Plant Ontology [14] provides a cell type node that shares some of the organizing principles of our cell ontology, but it is limited to those cell types found in plants. For all these reasons, we set out to produce an organism-independent ontology of cell types based on their properties (such as functional, histological and lineage classes) and report here the availability on the Open Biological Ontologies site [7] of this ontology, which incorporates the cell types possessed by a broad range of phyla and is defined by a rich set of criteria.

## Results

### The ontology

The first design decision was whether we should attempt to integrate cell types from all phyla within a single ontology or build independent ontologies for different taxonomic groups. The former has the great advantage of facilitating *de facto *integration of data from diverse databases, as described above. This approach does, however, pose conceptual problems: for example, are a mammalian 'muscle_cell' and a nematode 'muscle_cell' homologous? In this particular example we have little doubt that the answer is 'yes'; both of these cell types are evolutionary descendants of the first metazoan's 'muscle_cell'. In other cases, however, matters are not quite as straightforward, a plant 'hair_cell', a 'hair_cell' of the mammalian cochlea and an insect 'hair_cell' are probably not homologous, despite some similarities in their functions and genes expressed within them [15]. Despite these problems in building an 'integrated' cell-type ontology, the advantages, were we to succeed, outweigh them, and we have therefore taken this approach to develop a single ontology that integrates cell types from different phyla.

The ontology consists of concepts or terms (nodes) that are linked by two types of relationships (edges). This means that the ontology appears as a complex hierarchy (technically known as a directed acyclic graph, or DAG) where a given term (or concept) may not only have several children, but also several parents. The parent and child terms are connected to each other by *is_a *and *develops_from *relationships. The former is a subsumption relationship, in which the child term is a more restrictive concept than its parent (thus chondrocyte *is_a *mesenchyme_cell). The latter is used to code developmental lineage relationships between concepts, for example that a hepatocyte *develops_from *a mesenchymal_cell. The *is_a *relationship implies inheritance, so that any properties of the parent concept are inherited by its children; the *develops_from *concept carries no inheritance implications.

The rules for building the ontology are the same as those defined by the GO Consortium. That is, each concept in the Cell Ontology has an identifier with the syntax CL:nnnnnnn, where nnnnnnn is a unique integer, and CL identifies the Cell Ontology, (concepts should always be cited with their full identifier when being used in the context of a database). In addition, if there are precisely equivalent terms in other databases, for example in the Fungal Anatomy [16], *Arabidopsis *[17], Plant Ontology [14] or FlyBase databases [3], then the unique identifiers from these databases are included in the Cell Ontology. Most concepts in the Cell Ontology are provided with free-text definitions and may have one or more synonyms. Within the context of this ontology, synonyms are precise; a concept and its synonym can be exchanged without changing the concept's meaning. We use the same stratagem as does the GO when we have concepts that are lexically identical but have different meanings in different communities [18]. Thus, it is far from obvious that vertebrate and invertebrate pigment cells are homologous and these concepts are therefore described as pigment_cell_(sensu_Vertebrata) and pigment_cell_(sensu_Nematoda_and_Protostoma, respectively.

The two top-level nodes of the Cell Ontology are cell_in_vivo and experimentally_modified_cell. The former includes cell types that occur in nature, the latter those that are experimentally derived, including cell lines and such constructs as protoplasts. Experimentally derived cells are under-represented in the current version of the ontology. Naturally occurring cells are classified both by organism-independent categories and by organism (animal cells, plant cells, prokaryotic cells). The organism-independent classification of cells follows several different criteria that include: 'function' (for example, electrically_excitable_cell, secretory_cell, photosynthetic_cell), histology (for example, epthelial_cell, mesenchyme_cell), lineage (for example, ectodermal_cell, endodermal_cell) and ploidy (for example, haploid_cell, polyploid_cell). The present version of the Cell Ontology has an average 'depth' of about 10 nodes.

**Figure 1:**
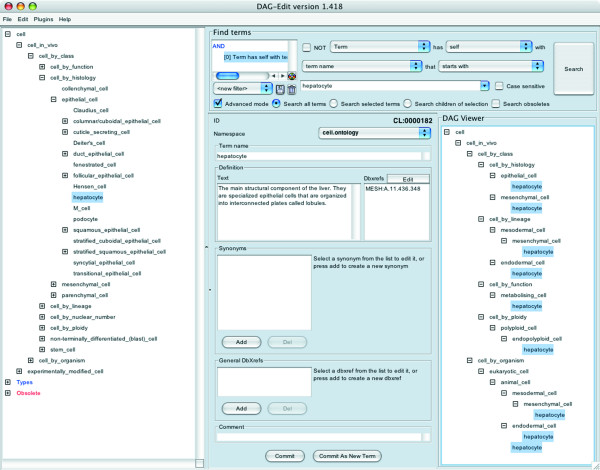
A screenshot of the cell ontology, as seen within the OBO-Edit program, displaying all the information associated with the term hepatocyte. The left-hand panel shows all the top-level terms, together with the location of hepatocyte within the cell_by_histology classification. The right-hand panel shows all the hierarchies within which hepatocyte can be found. The top part of the central panel illustrates how the term is found, while the lower part gives the definition, the unique Cell Ontology ID and the MESH ID.

The richness of the ontology can be illustrated by example (Figure 1). Kupffer cells are specialized vertebrate macrophages of the reticuloendothelial system. They function to filter small foreign particles (including bacteria) and old reticulocytes from the blood. In the Cell Ontology they are to be found by their function (they are a type of defensive_cell), by their lineage (they are derived from a mesodermal_cell derived from a hematopoietic_stem_cell, itself a type of stem_cell), by their morphology (they are a type of circulating_cell) and by their organism (they are a type of animal_cell).

## Discussion

Ontologies in bioinformatics are intended to capture and formalize a domain of knowledge, and the ontology reported here attempts to do this within the domain of cell types. It is designed to be useful in the sense that a researcher should be able to find, in a rapid and intuitive way, any cell type in any of the major model organisms and, having found it, learn a considerable amount about that cell type and its relationships to other biological objects.

A core feature of the ontology, and one that differentiates it from other resources that contain cell types such as SNOMED and the FMA [8,9], and the *Drosophila *and *Arabidopsis *ontologies [3,17], is that the cell ontology explicitly sets out to include cell types from all the major model organisms within a common framework. In addition, it also seeks to incorporate a great deal of phenotypic information about these cell types and is thus far more comprehensive in its cellular detail than these other resources. The intention is that the new cell-type ontology should provide organism-independent knowledge as well as cell-type unique identifiers (ID) that can be incorporated into any database holding cell-type-associated knowledge. The formalized structure of the ontology, together with its set of unique IDs, will allow curators to incorporate cell-type data into their databases, integrate the data with the knowledge encapsulated in the ontology, and use the IDs to interoperate with other databases. While we expect such bioinformatics applications to be its immediate use, we hope that, in the longer term, all biologists will find the ontology useful.

The expected short-term use of the ontology will thus be in cataloguing phenotypes and gene expression patterns. Indeed, it is quite surprising that those who work with model organisms still lack the bioinformatics resources needed to catalogue, archive and access the details of the phenotypes emerging from mutant screens and natural variations. A robust representation of normal and mutant phenotypes in all of the model organisms will require ontologies for a wide range of macroscopic properties (pathology, anatomy, abnormal quantifiers, and so on) and we view the cell ontology as a component of this programme that should be useful in cataloguing phenotypes (and other attributes) associated with cell types.

In the long term we expect that molecular biology and biological databases will move beyond being gene-centric and that biological mechanisms will be studied at a more integrated level. Cells are the biological units with which tissues and organs and organ systems are built. A rich and explicit description of cell types across phyla that are adapted by biological databases will help facilitate this transition.

Finally, it should be pointed out that, like many such resources, this ontology is not complete: although it contains all the common cell types, there will certainly be some that have been omitted. Most importantly, although many of the cell types are fully described by function, morphology, organism, and so on, others are inadequately described and more relationships need to be made. A particular weakness is the fact that the category identified as experimentally_modified_cell has yet to be populated, and doing this will involve consideration of the various cell lines held in the major collections. As with other community resources, community input is essential for the development and maintenance of the Cell Ontology; biologists with comments and additions are therefore welcome to contribute to the ontology and should contact the curator ashburner@ebi.ac.uk.

## Materials and methods

The ontology includes the major cell types from the major model organisms (for example, human, mouse, *Drosophila*, *Caenorhabditis*, zebrafish, *Dictyostelium discoideum*, *Arabidopsis*, fungi and prokaryotes). These cell types have been collated from our own knowledge, from major textbooks (for example [20-22]), from the embryo and anatomy ontologies available on the OBO site [7], and from colleagues (who are thanked in the acknowledgements). The ontology currently holds some 680 cell types, together with their synonyms and, in most cases, text definitions.

The ontology was constructed using the open source Java tool OBO-Edit (previously known as DAG-Edit) [23], which is convenient for building ontologies that are consistent with the GO formalism. The resulting ontology is available in both the GO 'flat-file' format [24] and the newly defined 'OBO format' [25], and can easily be viewed using the OBO-Edit or the COBrA open source Java tool [26].

## Availability

The Cell Ontology is available from the OBO site [19]. Following the cell.obo link will take the user to a page in which the current version of the Ontology, and archived older versions, can be viewed (view) or downloaded (download). Differences between the current and previous version can be seen by following the Diff to link.

## References

[B1] Mouse Genome Informatics. http://www.informatics.jax.org.

[B2] Maize Genetics and Genomics Database. http://www.maizegdb.org.

[B3] FlyBase. A Database of the Drosophila Genome. http://www.flybase.org.

[B4] Mammalian Phenotype Browser. http://www.informatics.jax.org/searches/MP_form.shtml.

[B5] Drysdale R (2001). Phenotypic data in FlyBase.. Brief Bioinform.

[B6] The Gene Ontology Consortium (2001). Creating the gene ontology resource: design and implementation.. Genome Res.

[B7] OBO: Open Biological Ontologies. http://obo.sourceforge.net.

[B8] SNOMED International. http://www.snomed.org.

[B9] Digital Anatomist Foundational Model. http://sig.biostr.washington.edu/projects/fm/AboutFM.html.

[B10] ATCC: The Global Bioresource Center. http://www.atcc.org.

[B11] European Collection of Cell Cultures. http://www.ecacc.org.uk.

[B12] Cell Type. http://www.sanbi.ac.za/evoc/ontologies_html/latest/celltype.html.

[B13] Tissue DB. http://tissuedb.ontology.ims.u-tokyo.ac.jp:8082/tissuedb.

[B14] Plant Ontology Consortium. http://www.plantontology.org.

[B15] Kiehart DP, Franke JD, Chee MK, Montague RA, Chen T-L, Roote J, Ashburner M (2004). *Drosophila crinkled*, mutations of which disrupt morphogenesis and cause lethality, encodes fly myosin VIIA.. Genetics.

[B16] Fungal Anatomy Ontology Project. http://www.yeastgenome.org/fungi/fungal_anatomy_ontology/#description.

[B17] TAIR. The Arabidopsis Information Resource. http://www.arabidopsis.org.

[B18] Gene Ontology. GO Editorial Style Guide. http://www.geneontology.org/GO.usage.html#sensu.

[B19] SourceForge.net CVS repository. http://cvs.sourceforge.net/viewcvs.py/obo/obo/ontology/anatomy/cell_type.

[B20] Williams PL, (Ed) (1996). Gray's Anatomy.

[B21] Alberts B, Johnson A, Lewis J, Raff M, Roberts K, Walter P (2002). The Molecular Biology of the Cell.

[B22] Esau K (1977). The Anatomy of Seed Plants.

[B23] SourceForge.net Project File List. http://sourceforge.net/project/showfiles.php?group_id=36855.

[B24] GO documentation: file format guide. http://www.geneontology.org/GO.format.html.

[B25] Gene Ontology: the OBO flat file format. http://www.geneontology.org/GO.format.html#oboflat.

[B26] XSPAN. A cross species anatomy network. http://www.xspan.org/applications/cobra/index.html.

